# Quantum Ninjas vs Laser Robots: A Board Game to Cultivate Quantum Intuition

**DOI:** 10.1021/acs.jchemed.5c01888

**Published:** 2026-09-04

**Authors:** Amirhosein Amini, Pritha Ghosh, Logan Blackham, Arshath Manjalingal, Saeed Rahmanian Koshkaki, Sachith Wickramasinghe, Elmo J. Mawk, Joshua D. Edwards, Soon-Mi Lim, Arkajit Mandal

**Affiliations:** Department of Chemistry, 14736 Texas A&M University, College Station, Texas 77840, United States

**Keywords:** Upper-Division Undergraduate, Physical Chemistry, Games, Hands-On Learning, Analogies, Quantum Chemistry

## Abstract

In this work, we introduce *Quantum Ninjas vs Laser Robots*, a board game designed to cultivate quantum intuition in learners across a variety of backgrounds through interactive gameplay and tangible analogies. The game introduces familiar and approachable characters, ninjas and robots, which help to model key ideas of quantum mechanics, including probabilistic measurement, discrete energy levels, excitation and relaxation processes, and wave function collapse. The game was introduced to a group of undergraduate students at Texas A&M University, and their postgame perceptions were collected via a survey. The results indicate a positive reception of the game, engagement, and collaborative learning. Our findings suggest that game-based learning has the potential to serve as an effective complement to traditional teaching methods for abstract and math-heavy subjects such as quantum mechanics by lowering barriers to entry.

## Graphical abstract

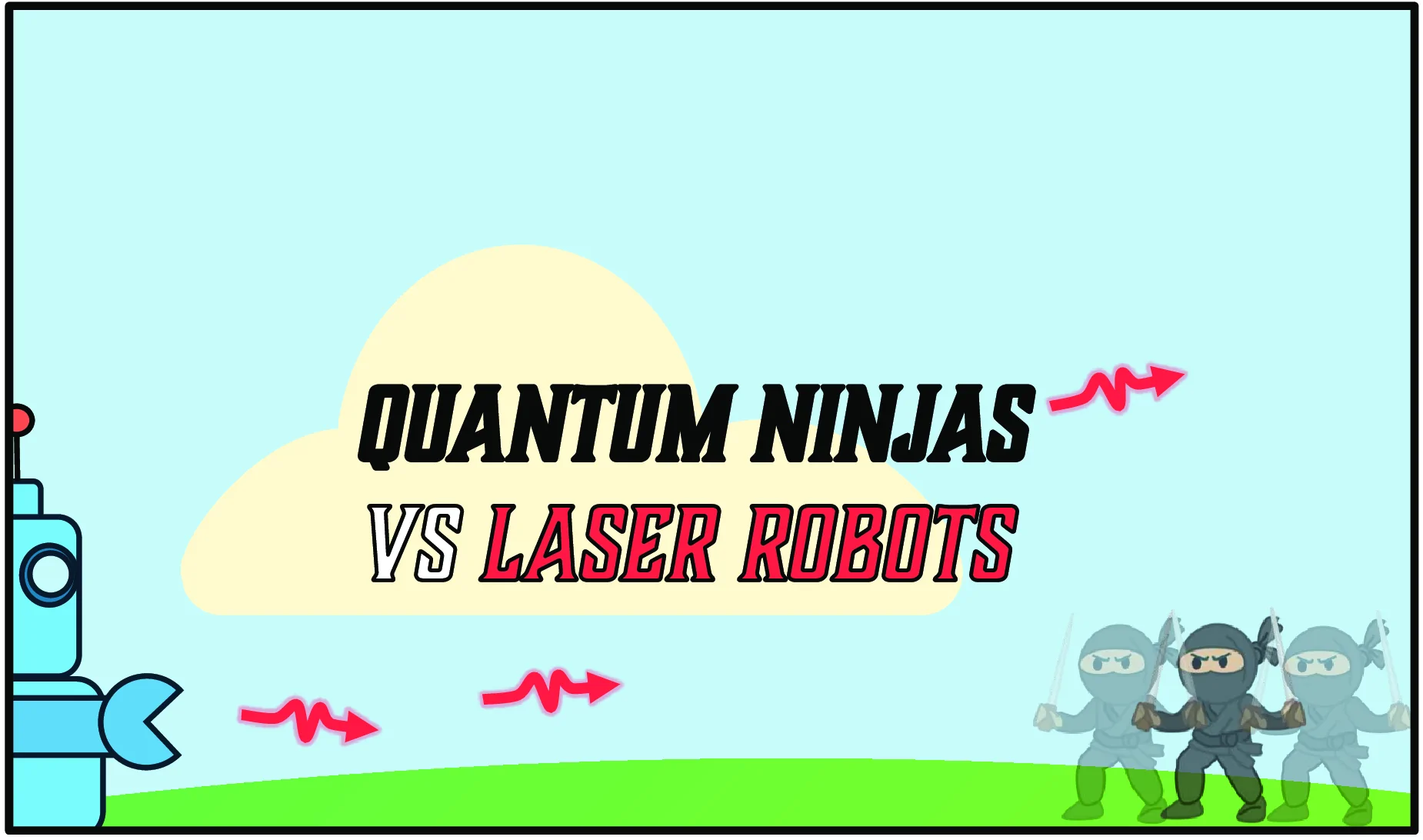

## 1 Introduction

Classical mechanics can be taught using real-world experiences and hands-on experiments. Quantum mechanics, on the other hand, is fundamentally counterintuitive, and the disconnection from lived, real-life experiences makes it a difficult subject to teach.^1–4^ In addition to this, most students rarely achieve the mathematical proficiency required for learning quantum mechanics by the end of high school, and this gap persists even among undergraduate students.^5^ The mathematically rigorous understanding of quantum mechanics requires a solid foundation of linear algebra and differential calculus, topics that present as a barrier to learning and deter students from engaging the subject. To address this gap, *gamification* provides an alternative entry point into quantum concepts that reduces these barriers by transforming abstract concepts into accessible, tactile, and interactive experiences that engage them through play, experimentation, and peer interaction.^6,7^

More recent chemical education publications have introduced interactive simulations, video games and immersive visualization tools to make abstract physical chemistry concepts more approachable.^1,8–10^ These include designing interactive simulations to introduce quantum mechanics,^1^ making a board game to review Physical Chemistry I concepts,^10^ and digital and immersive tools which enable learners to visually learn about concepts such as molecular orbitals, potentials, molecular dynamics and time-dependent Schrödinger equation.^8,9^ These studies show the growing role of interactive teaching strategies in chemical education, through accessible and engaging media, such as boardgames and video games.

Consequently, previous work has been focused on creating ”quantum games” that aim to make challenging concepts more accessible for students.^6,7,11^ One such game is *Quantum Tic-Tac-Toe*,^12^ which uses a modified version of the classic tic-tac-toe game to teach concepts such as superposition, entanglement, and measurement. Another example is *Quantum Minesweeper*,^13^ which is designed to highlight the differences between classical and quantum measurements, among other concepts.

In the game introduced here, *Quantum Ninjas vs Laser Robots*, we aim to emphasize the probabilistic nature of quantum mechanics and how it depends on the quantum mechanical state of a particle, an aspect that has not been thoroughly explored in previous quantum games. The game consists of a board with five quantum states, |0⟩, |1⟩, |2⟩, |3⟩, |4⟩ (representing discrete energy levels), a ninja (representing a quantum particle), and three laser robots (representing measurement devices). Each ninja has four photon coins and one emission card. Each photon coin allows the ninja to move one level up, while the emission card returns it to the ground state |0⟩. The ninja starts in the ground state; the robots choose their measurement positions at the bottom of the board and then roll a 20-sided die to determine whether they successfully catch the ninja, based on the outcome of the roll. During the game, the player controlling the ninja can use the photon coins and the emission card to jump between different states to increase their chances of survival, while the robots can change their location to increase the probability of capturing (i.e., collapsing the wave function) the ninja. Actions in the game aim to conceptualize real quantum mechanical processes such as measurement, probability, excitation, and emission.

Our goal is to provide an intuitive feel for the probabilistic nature of quantum mechanics through a game in which winning or losing depends not only on a player’s skill but also on a probability distribution. The game is designed to be adaptable across different audiences, including middle and high school students, members of the general public, and even graduate students. It should be noted, however, that this study only reports implementation in an undergraduate physical chemistry laboratory course. Players are not required to engage with the complex mathematical foundations of quantum mechanics in order to grasp its basic concepts, thereby reducing the intimidation that often accompanies quantum mechanics.

## 2 Physical Concepts and Gameplay

### 2.1 Ninja and the Wave-Particle Duality

All quantum particles exhibit wave-particle duality, meaning each quantum particle simultaneously exhibits both wave-like and particle-like behavior, which is a central concept in quantum mechanics. This dual characteristic, seen at submicroscopic (“quantum”) scales and not exhibited by everyday objects that we typically interact with, is not only challenging to visualize but also to comprehend due to the abstract nature of the concept itself.

To playfully introduce this concept, we introduce the ninja, a central game piece of the board game introduced here, with a *special ability*: the power to create shadow clones—illusory copies that exist in a spatially delocalized fashion, suggesting its presence in more than one location at the same time. This ability directly resonates with the wave-like spatially delocalized nature of quantum particles. Our inspiration comes from the *shadow clone technique* ^14^ (Bunshin no Jutsu) exhibited by fictional ninja characters (such as in “Naruto”, “Ninja Hattori” and “Dragon Ball Z”), popular in Japanese animation and easily recognizable by teenagers, kids and young adults.

“Shadow cloning” allows ninjas to create one or more illusory copies of themselves, making it difficult for an opponent to make a precise attack on the ninja. This idea maps onto the (delocalized) behavior of a quantum particle, which does not possess a definite position in space but is instead described by a probability distribution function, which is just the squared amplitude of its ”wave function”.^15^ An undisturbed quantum particle therefore behaves like a wave, spread out over space. However, when a measurement is performed to detect it, the particle is found at a single location, effectively collapsing into a particle-like form, like popular fictional ninja characters in various animated cartoons. We elaborate on this in the next section.

### 2.2 Rolling a Die and Wave Function Collapse

The measurement postulate of quantum mechanics^16^ states that a projective measurement collapses a quantum state, and the probability of each outcome is given by the Born rule (as in the Copenhagen interpretation^17^). This is often compared to a coin toss, but that analogy is misleading: a coin has two *equal* outcomes, whereas quantum measurement outcomes generally have *unequal* probabilities set by the squared amplitudes of the wave function.

In our board game (Figure 1), the ”ninja” can occupy one of five discrete quantum states |ψ⟩∈{|0⟩, ···, |4⟩}, each associated with a spatial probability *p*_*k*_ ∝ |ψ­(*x*_*k*_)|^2^ = |⟨*x*_*k*_|ψ⟩|^2^ where *k* = 1, 2, ···, 10 such that the position space is discretized into *N*_*x*_ = 10 grid points. To be more specific, the normalized Born probabilities are

$$p_{j}=\frac{|\psi \left(x_{j}\right){|}^{2}}{\overset{N_{x}=10}{\underset{k=1}{\sum }}|\psi \left(x_{k}\right){|}^{2}},\overset{N_{x}=10}{\underset{j=1}{\sum }}p_{j}=1$$

**Figure 1:**
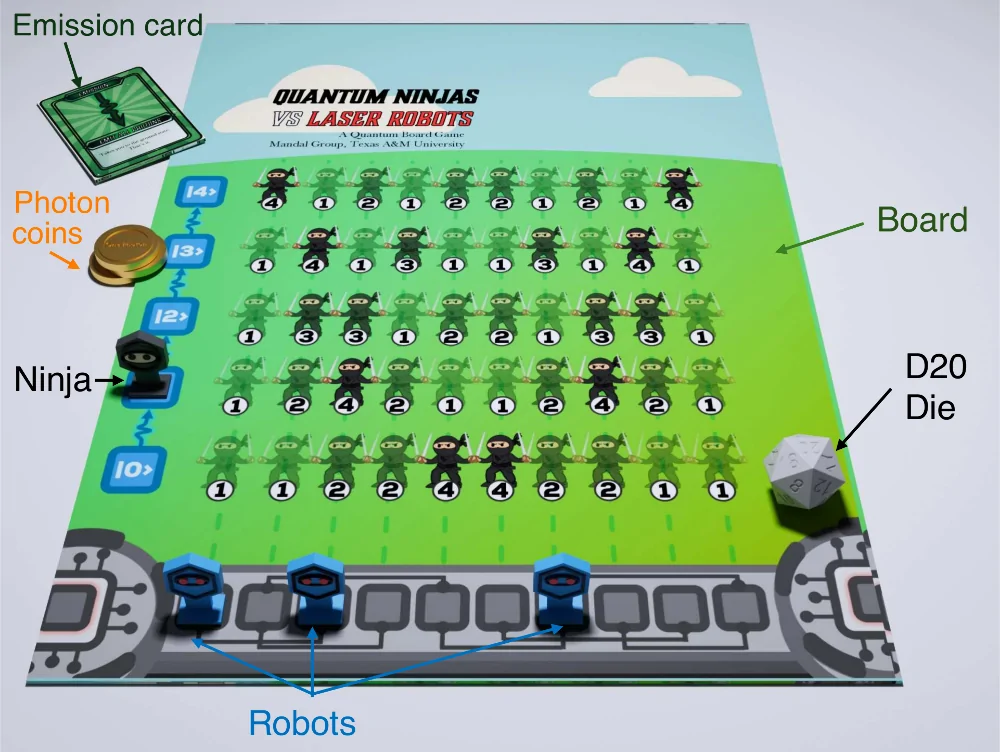
Board layout of *Quantum Ninjas vs Laser Robots*. Robots are placed in the gray boxes at the bottom of the board, and the ninja is placed in one of the blue boxes on the left, each representing an energy level. The game also includes a 20-sided die, photon coins and an emission card.

To ”simulate” a projective measurement (collapse) using a 20-sided die, we define an unnormalized probability *P̃*_*j*_ = 20*p*_*j*_ so that ∑ _*j* = 1_^10^ *P̃*_*j*_ = 20. Let α ∈{1, 2, ···, 20} be the outcome of a die roll, then the wave function collapses to state *j* if α ≤ ∑_*j*∈**R.P.**_ *P̃*_*j*_ where the summation is over the three robot positions (R.P. means “Robot Positions”). For α > ∑_*j*∈**R.P.**_ *P̃*_*j*_, the ninja evades capture. The values of *P̃*_*j*_ are tabulated on the board (see Figure 1).

The probability patterns were inspired by the qualitative spatial structure of the lowest one-dimensional harmonic oscillator states. For gameplay, each continuous spatial distribution was simplified onto ten spatial positions and represented by integer weights from 1 to 4. The weights were chosen so that the values in each energy level sum to 20, allowing the corresponding probability distribution to be sampled directly using a 20-sided die.

This procedure makes clear that measurement outcomes are both probabilistic *and* generally unequal across different locations. Repeating the measurement many times will empirically reproduce the distribution {*p*_*j*_}, reinforcing ideas such as electron-density “clouds” and the probabilistic interpretation of atomic and molecular orbitals that is fundamental to quantum chemistry and quantum physics.

### 2.3 Photon Coins and Quantization

A fundamental principle in quantum mechanics is the discreteness of energy, which helped explain various fundamental phenomena such as the ultraviolet catastrophe in blackbody radiation and the photoelectric effect. These discoveries eventually led to the establishment of quantum mechanics. It is therefore important for learners to develop a solid understanding of this principle early in their quantum journey. Despite the classical, continuous picture of energy that can be taught through hands-on experiments, it is not easy to introduce this quantum phenomenon to learners because there are few tangible real-life analogies for it.

To make this rather abstract idea more tangible, we represent photons as physical “photon coins”. Each coin corresponds to a single *quantum* of energy. Players must spend the appropriate number of photons to move the ninja to a higher level which directly maps to the similar requirement for quantum particles to move between different discrete energy levels. This analogy helps reinforce two important concepts: the ninja can only exist at certain discrete levels and can only move between them by spending these “photon coins”. With the help of this analogy, learners build an intuitive sense for the concept of quantized energy and the role of photons in enabling quantum particles to move between the allowed energy levels.

### 2.4 Gameplay

The game has two players. One player controls the robots, whose goal is to detect and capture the ninja. The other player controls the ninja, who has the ability to move between different energy levels to avoid being captured. Each round follows the same sequence of three steps detailed below.

#### 2.4.1 Step 1 - Robot Placement

The robot player is in control of three robot markers (see Figure 1). At the start of each round, the player places the robots in any three slots at the bottom of the board, these positions represent where the measurement devices are positioned. At this point, the player controlling the robots has no information on where the ninja is located.

#### 2.4.2 Step 2 - Ninja Movement

The ninja player starts at the ground state, marked with a |0⟩ on the board. The ninja has a limited set of single-use resources: 4 photon coins which allow it to move up the energy levels and 1 emission card that allows it to relax back to the ground state, no matter where the ninja is located on the board. During this step of each round, the ninja player may remain at the current energy level without using a resource, spend one or more available photon coins to move upward by one energy level per coin, or use the emission card to return to the ground state from any energy level. Because the ninja has only 5 possible moves during the game, the player must think strategically based on the positions of the robots: avoid being captured while still leaving options for later rounds.

#### 2.4.3 Step 3 - Dice Roll

After the ninja occupies an energy level or decides to remain in the ground state, the robot player rolls a 20-sided die. Every position on the board is labeled with an integer, which all add up to 20 for all energy levels. If the outcome of the roll is less than or equal to the sum of the numbers to which the robot player is pointing, the ninja is captured, and the robot player wins the round. If the roll results in a higher number, the ninja successfully survives the attack. For example, for the placement of robots shown in Figure 1, a dice roll of 7 (1 + 4 + 2) or less leads to the collapse (and capture) of the ninja, while a dice roll of 8 or larger means the ninja avoids being captured. In this example, Figure 1 depicts the board after the ninja has moved to |1⟩ and immediately before the die roll.

These three steps are repeated for **four** rounds; if the ninja gets caught during these four rounds the robots win; otherwise, the ninja is successful in evading capture and thus it wins.

#### 2.4.4 Quantum Analogy

Each part of the gameplay aims to mirror a key idea in quantum mechanics. The position of the robots corresponds to where the measurement devices are placed, while the ninja represents a quantum state that is about to be measured, and can only occupy select energy levels. The photon coins directly model absorption, spending energy to move to higher energy levels, while the emission card, as the name suggests, represents an emission and return to the ground state. The die roll plays the role of the measurement and its stochastic nature. Before the roll, the ninja’s location is uncertain from the observer’s point of view, as soon as the die is rolled, the superposition collapses to a definite outcome: the ninja is either captured or not, depending only on the probability distribution of the ninja in space. This setup models the wave function collapse and the probabilistic nature of quantum mechanics in a playful and engaging manner.

## 3 Results and Discussion

### 3.1 Implementation Details

The Quantum Ninjas vs Laser Robots game was implemented in Physical Chemistry Laboratory II (CHEM326) course at Texas A&M University in Fall 2025. The course comprises 4 modules. The first module is called “Computational Chemistry” and introduces students to quantum chemistry through 3 experiments. The first experiment introduces them to the basics of quantum chemistry, introducing the time-independent Schrödinger equation, the Born–Oppenheimer approximation, potential energy surfaces, Lennard-Jones and Morse potentials, and approximate methods for solving the electronic Hamiltonian like Hartree–Fock and second-order Møller-Plesset perturbation theory. The second and third experiments explore thermochemistry and frequency calculations for select organic molecules. The students are given access to an electronic structure software that helps them build molecules, modify xyz files, and run their calculations.

The game was introduced during the first module as a warm-up activity for quantum mechanical concepts that students would encounter later in the course. It should be noted that because the students were upper-level chemistry students, some may have previously encountered related concepts through concurrent lectures or earlier coursework. Before gameplay, students were told they will play a board game with the goal of easing the understanding of the concepts. The instructor gave a brief 5–10 min introduction to the gameplay by showing the game materials and explaining the role of each player. The instructor then demonstrated a sample round before asking the students to play the game. The students then were asked to play the game in pairs of 2 (one ninja and one robot player) plus a group of 3 (two students playing robots and one ninja player), when the number of students was odd. The activity was performed at the beginning of the first module and took approximately 30 min. The performance or survey responses of students were not used to grade this module. Our survey is designed to evaluate their overall understanding of fundamental quantum concepts which aligns with the learning objective of our physical chemistry lab. However, because no pregame assessment was performed, these results do not directly quantify learning gains of this board game. To make this activity easier to adopt in various settings, we have included a rule sheet which can be found in the Supporting Information.

Students answered anonymous surveys after playing the game. All recruitment and study procedures were approved by the university’s Institutional Review Board (IRB) with the IRB approval ID STUDY2025-0926. Due to the fact that the survey was administered only after gameplay and the lack of a pregame survey, the responses are interpreted as self-reported assessments and perceptions, rather than direct evidence of conceptual learning gains.

No unexpected or unusually high safety hazards were encountered during this activity.

### 3.2 Results

Figure 2 summarizes the responses of 27 students on the quantum mechanical concepts represented through the game. The results show a generally positive trend for all four statements. Moreover, it can be seen that agreement was strongest for the probabilistic nature of quantum measurement, description of every quantum particle by the wave function and its meaning and the collapse of the wave function (Figure 2(a,c,d)). However, the responses regarding the quantization of photon energy are somewhat more varied. Overall, the survey responses indicate that the participants generally recognized the conceptual connection intended by the game, although these results are self-assessed and do not independently demonstrate learning gains. That said, these results provide preliminary evidence that their responses are aligned with the learning objective of this module of the course.

**Figure 2:**
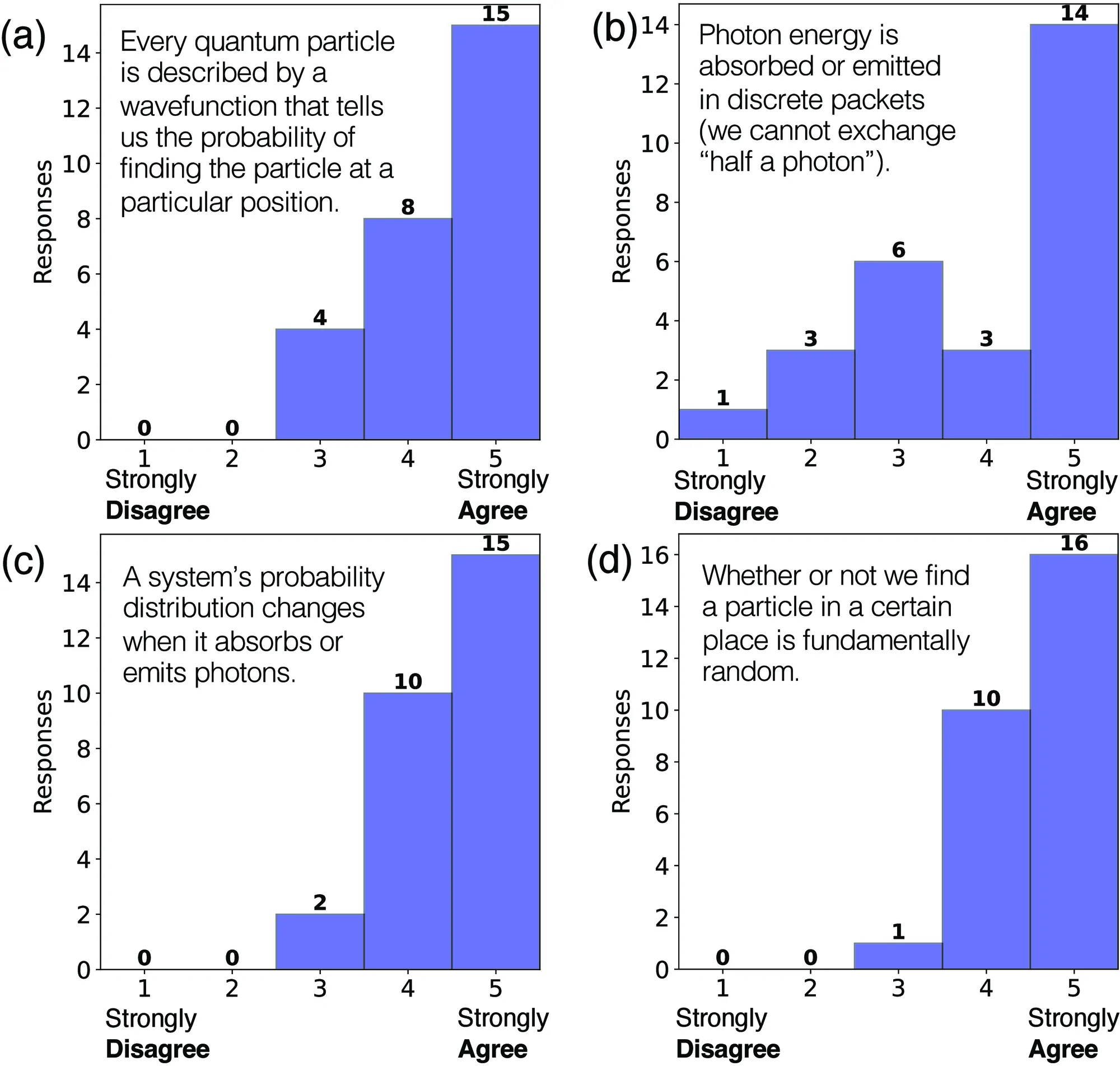
Postgame survey responses (*N* = 27). Histograms show levels of agreement with statements targeting quantum concepts: (a) the wave function as a probability description, (b) photon energy quantization, (c) changes in a system’s probability distribution upon absorption or emission, and (d) the inherent randomness of quantum mechanics.

Figure 3 summarizes students’ perceptions of their game-based learning experience. It can be seen that the students report varying levels of prior familiarity with the subject and how much confidence they have in their background knowledge. Most students agree that the instructions were clearly given, that the game connects to the quantum-mechanical concepts and that this activity provided a sense of belonging. Moreover, many participants report increased familiarity, improved understanding, and perceived helpfulness after playing the game; however, it should be noted that these responses represent the students’ impression, rather than direct learning measurement.

**Figure 3:**
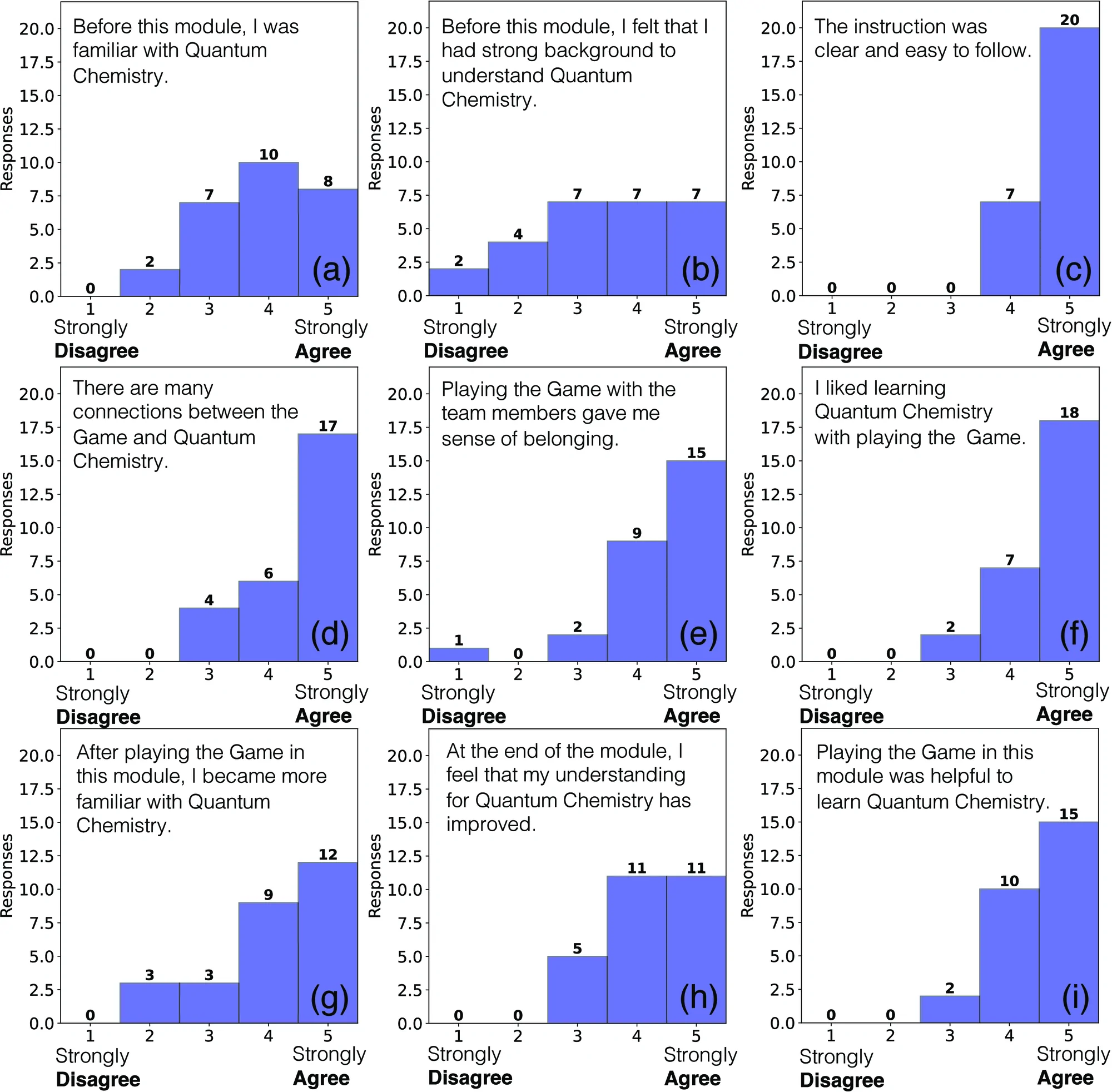
Self-reported student feedback on the game-based learning experience (*N* = 27). Panels show responses regarding (a) and (b) prior knowledge about quantum mechanics, (c) clarity of game instructions, (d) connections between the game and quantum mechanics, (e) sense of belonging during gameplay, (f) enjoyment of learning quantum mechanics through the game, (g) increased familiarity after playing, (h) improved understanding by the end of the module, and (i) overall helpfulness of the game for learning quantum chemistry.

Our results are limited by the absence of a pregame survey, the possible prior exposure of students to quantum mechanics, and the limited number of participants. Future studies would benefit from validated pre- and postgame surveys and a more diverse group of participants.

## 4 Conclusion

In this work, we introduced *Quantum Ninjas vs Laser Robots*, a board game aimed at cultivating quantum mechanical intuition and facilitating its learning through an interactive and engaging gameplay. Through this game we introduce several key quantum mechanical concepts including: wave-particle duality, the delocalized nature of quantum particles, discrete nature of energy, and the probabilistic nature of the wave function collapse.

The survey results, obtained from a group of undergraduate students at Texas A&M University, show that the students reported positive experiences and learning benefits of these key concepts in quantum mechanics by playing Quantum Ninjas vs Laser Robots. The game features a ninja representing a quantum particle, measurement devices in the form of laser robots, and photon coins to build a tangible understanding of discrete energy levels. We show that this approach can be used as a promising approach to introduce abstract quantum concepts with playful, hands-on interactions.

Overall, our game fosters a sense of belonging and cooperative learning, with self-reported positive perceived learning benefits from students familiarity and understanding of quantum mechanics underscores its potential as a valuable educational tool. Our survey results illustrate that the students reported a better understanding and greater engagement, suggesting that the present game can be an effective tool for introducing quantum mechanics to students. The present game can easily be used in different settings, such as classrooms and science outreach events.

In conclusion, *Quantum Ninjas vs Laser Robots* demonstrates the potential of game-based learning to cultivate quantum intuition. Our future plans include further refinements of the gameplay to enhance the learning experience. We also plan to develop a newer version of the board game (and a browser-based version of the game) that includes concepts such as superposition of different states, entanglement, and decoherence. Overall, we envision that our game presents a creative solution that will help resolve the longstanding challenge of making quantum mechanics more intuitive, approachable, and accessible to a broader audience.

## Supplementary Material



## Notes

Rule sheet, printable game board and tokens, 3D-printable files for the physical game pieces, and a supplemental video explaining the rules and demonstrating gameplay (ZIP)
