# Supplementary material for: Quantum Ninjas vs Laser Robots: A Board Game to Cultivate Quantum Intuition
Source: J Chem Educ. 2026 Sep 4;103(10):6084–9. doi: 10.1021/acs.jchemed.5c01888 (PMC13674001; doi:10.1021/acs.jchemed.5c01888)
Supplement: Supplementary file 1 [file ed5c01888_si_001.zip › 2D Materials/Emission_Card_Side1.pdf]

EMISSION

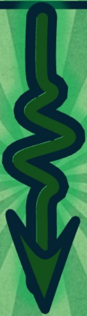

EMIT ALL PHOTONS

Takes you to the ground state.
That’s it.
