# Supplementary material for: Quantum Ninjas vs Laser Robots: A Board Game to Cultivate Quantum Intuition
Source: J Chem Educ. 2026 Sep 4;103(10):6084–9. doi: 10.1021/acs.jchemed.5c01888 (PMC13674001; doi:10.1021/acs.jchemed.5c01888)
Supplement: Supplementary file 1 [file ed5c01888_si_001.zip › 2D Materials/RuleSheet.pdf]

QUANTUM NINJAS VS LASER ROBOTS

A Quantum Board Game

Mandal Group, Texas A&M University

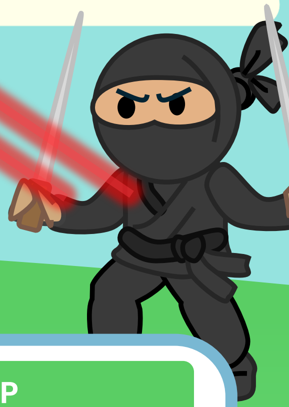

OBJECTIVE

- The robot player wins if they capture the ninja at any round.
- The ninja player wins by surviving 4 rounds.

GAME PIECES

- 1 board
- 3 robots
- 1 ninja
- 1 d20 die
- 4 photon coins
- 1 emission card

SETUP

1- Place the ninja token at |0›.

2- The ninja player gets 4 photon coins and 1 emission card.

3- The robot player gets 3 robot tokens.

GAMEPLAY

STEP 1: Place the robots

The robot player places three robots in any of the 10 bottom slots.

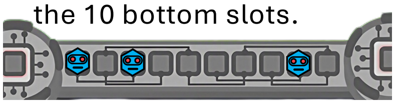

STEP 2: Move the ninja

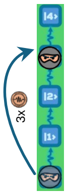

The ninja may stay where it is, move up by spending photon coins or relax to |0› by using the emission card.

STEP 3: Roll the die

Roll the d20. If the roll is less than or equal to the sum of the robot-marked numbers, the robots win; otherwise, the game continues to the next round.

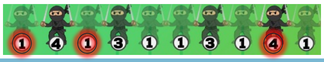

AN EXAMPLE ROUND

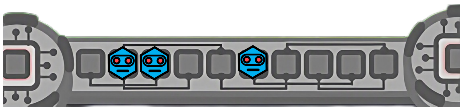

→

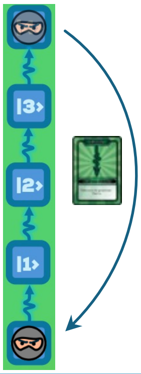

→

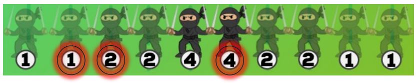

1 + 2 + 4 = 7

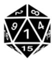

Roll 1-7	Roll 8-20
**NINJA CAPTURED!**	**NINJA SURVIVES!**
